# Hospital-based Palliative care: A Case for Integrating Care with Cure

**DOI:** 10.4103/0973-1075.76248

**Published:** 2011-01

**Authors:** Priya Darshini Kulkarni

**Affiliations:** Cipla Palliative Care and Training Centre, Pune. Maharashtra, India

**Keywords:** Hospital based palliative care, Progress of Palliative care in India, Comprehensive care ofpatients

## Abstract

The reason that probably prompted Dame Cicely Saunders to launch the palliative care movement was the need to move away from the impersonal, technocratic approach to death that had become the norm in hospitals after the Second World War. Palliative care focuses on relieving the suffering of patients and families. Not limited to just management of pain, it includes comprehensive management of any symptom, which affects the quality of life. Care is optimized through early initiation and comprehensive implementation throughout the disease trajectory. Effective palliative care at the outset can help accelerate a positive clinical outcome. At the end of life, it can enhance the opportunity for the patient and family to achieve a sense of growth, resolve differences, and find a comfortable closure. It helps to reduce the suffering and fear associated with dying and prepares the family for bereavement.

## HEALING IN WHOLE

Taking the example of cancer, modern medicine has made several new promising cancer treatments available for many cancers today. As a result, more and more people with the incurable disease are living longer. However, that has also made cancer a chronic illness, with many patients living lon-ger with symptoms. These survivors have different physical, social, psychological, economic, and even legal concerns.

Despite the advances in therapy, cancer remains a devastating illness-for the patients, for their families, the community, and health care providers. Talking of treatment, there is always hope for cure, but one also hopes to be healed during the process. If one can not be cured, the patient can still die healed.

What is important is having a sense of wholeness as a person, at any stage of the disease. Philosophy of palliative care supports the patient’s search for normalcy, dignity, and comfort. Therefore, it is the responsibility of health care professionals to ensure that the patient with cancer or any life-limiting illness has a chance at being both cured and healed. This is possible when there is seamless integration of palliative care and acute care through-out the trajectory of the disease. This calls for collaborative care between oncologists and palliative medicine experts. Such shared management can maximize comprehension of patient’s distress and optimize support provided during hospitalization.

## INDIAN SCENE

While the integration of palliative care with curative therapies is already a reality in developed countries, India still has a long way to go. Palliative care has been developing in India since the mid-1980s. Palliative care teams, whether in the form of NGOs or other groups, have made important contributions in improving the care of patients with incurable illnesses in the country over the past 20 years. At any given point of time, around 2.5 million people suffer from cancer in India. Every year, almost two lac new cancer cases are diagnosed, and 70% percent of these cases are detected in advanced stages. Of these cases, only 1.6 million people get any kind of treatment and only 0.4% receives relief through palliative care. With other chronic debilitating illnesses, like HIV, chronic renal failures, geriatric conditions, it becomes clear that compared to the magnitude of the need, only a minuscule proportion of the needy actually receive palliative care. There is an urgent need to introduce palliative care in primary, secondary, and tertiary health care facilities.

Organizations like Indian Association of Palliative Care, Pallium India and the Institute of Palliative Medicines are working with an increasing number of individuals and associations to further palliative care. There has been a significant change in the mindset of health care providers, and policy makers with respect to the urgency in providing palliative care. Palliative care services have acquired an increasing role in incurable diseases including cancer. But, palliative care is not yet included in the undergraduate curriculum and not accepted as a speciality by the Medical Council of India. It is still regarded as depressing, uninteresting, or of little intellectual value by medical professionals. It is often mistaken for end-of-life care.

## CASE FOR INTEGRATION

Out in the West, palliative care is no longer just tender loving care delivered toward the end of life but a legitimate medical speciality that seeks to enhance the effectiveness of curative treatment by controlling pain and other symptoms. The palliative care specialist is a member of the team responsible for a patient’s care right from the time of diagnosis. The competencies of palliative medicine are in relieving the suffering and promoting good quality of life for patients and families. The palliative medicine specialist, with his multidisciplinary team, adds a unique perspective in assessing and treating pain and other symptoms, and conveys clarity in ethical decision making for patients with advanced disease. Proficiency in symptom control, developed over many years in the palliative setting, has proven to be adaptable to patients in the acute-care setting.

Contributions by palliative medicine specialist in symptom control, decision making, management of treatment complications, communication, psychosocial care, and coordination of care have resulted in improved care of terminally ill hospitalized patients. When these patients are discharged from the hospitals, appropriate referrals are made to community-specialized services. This assures continuity of care when the patient is shifted from acute hospital to community palliative care service and enables the full spectrum of services for patients and their families from the time of diagnosis throughout the course of the illness.

In India, most patients with cancer and other terminal illnesses are diagnosed and treated in acute hospitals. Acute hospitals are also the most common setting, where people die. Thus, there is a need for skilled and compassionate care of the dying in the acute hospital setting. It is necessary that medical professionals who care for the dying on a day-to-day basis must have training in palliative care. This is important, given the unpredictability of the terminal phase of diseases. Admission to a remote palliative care unit becomes difficult given the unpredictable prognosis. Formal recognition of palliative medicine as a speciality would help people realize that palliative care too is a relevant goal of medicine when it comes to dealing with life-limiting illness. So, there is a dire need for collaboration of palliative medicine with other specialties, allowing communication and exchange of ideas on issues relevant to the medical care of patients with incurable disease.

There are proven benefits of integrating palliative services with hospitals. By including palliative services, hospital staff benefit from on-the-job training in end-of-life care and critical communications. Palliative care team can take advantage of the organizational resources of the hospital to provide greater care to a larger number of people. Serious and terminally ill patients can get better quality of holistic care covering social, spiritual and psychological aspects. The hospital gets an enhanced positive image in the community for providing better care that encompasses not only the patient, but also the relatives. Sometimes there is reduction in cost of care through shortened duration of stay and patient-specific care.

Such a partnership may bridge acute care and palliative care, while multiplying the strengths and eliminating the limitations of both. With current and updated knowledge, there is continuity of care that enhances the ability of both partners to tailor care plans to meet patient and family needs and preferences. Hospitals may contribute to the partnership by acute care expertise across multiple specialities, management and marketing capabilities, and library and information system resources. Palliative care centers can contribute to the partnership by facilitating advance care planning, end-of-life care clinical services, bereavement support, and volunteer training and integration. Thus, both partners bring strengths to the development and implementation of the optimum patient care program.

In India, a few cases of successful collaboration between palliative care centers and hospitals already exist. Now, more and more people are realizing the importance of palliative care. Despite stiff resistance from the majority, some oncologists have understood the importance of referring patients to palliative care early during the course of the disease. These professionals correctly perceive palliative care as an integral part of care from diagnosis to death, rather than being limited to the terminal phase. Palliative care is also expanding its scope beyond cancer care and beyond terminal care. At least in pockets, there are emerging subspecialties like geriatric palliative care, pediatric palliative care, etc.

So, in order to further palliative care services in India, all the specialities should come together and embrace palliative care as an approach, rather than a competitive speciality. Just as it is incorrect to perceive death as the “failure” of medicine, it is wrong to look at palliative care as the last optional step before death. For the sake of better health care, the focus should be on the whole-person quality of the patient and the family right from diagnosis to death and beyond. Shared decision making and advance care planning facilitated by integrated care can make this happen every single time, without any extra effort. Integrated care can serve patients without denying the inevitability of death and do all that is necessary to achieve relative comfort. Clinicians in such joint teams are in a better position to acknowledge and appreciate the lifelong human capacity for growth. And as many clinicians have acknowledged, they have been happily surprised by their own well-rounded growth, simply by being part of the team that helps discover afresh the value of dying within the mystery of life.

